# Treatment of the fresh central slip avulsion of the extensor tendon by bone tunnel-tendon suture: a prospective cohort study

**DOI:** 10.1186/s12891-021-04014-0

**Published:** 2021-02-04

**Authors:** Bin Wang, Yiming Lu, Tianliang Wang, Jiaxiang Gu, Naichen Zhang

**Affiliations:** 1 Clinical Medical College, Yangzhou University, Yangzhou, 225009 Jiangsu Province China; 2 Department of Foot and Hand Surgery, Northern Jiangsu People’s Hospital, Yangzhou, 225001 Jiangsu Province China

**Keywords:** Central slip avulsion, Tendon injury, Bone tunnel, Tendon-bone healing

## Abstract

**Background:**

The current evidence base for the management of central slip avulsions is limited from obtaining a best approach. The purpose of this study was to evaluate the clinical effect and feasibility of repairing the fresh central slip avulsion by bone tunnel-tendon suture.

**Methods:**

Twenty-four cases of open and closed central slip avulsions were prospectively studied. They were treated by suturing the tendon to the pre-holed bone through two parallel bone tunnels. Follow-up was conducted at 1 month, 3 months, 6 months, 12 months and 18 months after the operation. Symptoms, degree of satisfaction with the appearance, complications, Crawford’s evaluation, DASH scores and the total active movement (TAM) were collected.

**Results:**

The follow-up period was 6~18 months (mean 13 months). Finger function was assessed using the Crawford’s evaluation criteria: excellent in 12, good in 10, average in 2, with an excellent and good rate of 91.7%. DASH scores ranged from 37 to 47(mean 39). According to the Chinese Medical Association’s trial criteria for assessing the function of upper limbs, excellent, good and average cases were 9, 14 and 1 respectively. The range of motion gradually improved over time. **Conclusions** Good prognosis can be achieved through bone tunnel-tendon suture for the treatment of fresh central slip avulsion.

## Background

The central slip avulsion of the extensor tendon is a complex problem which may result in boutonniere deformity without in-time treatment [1]. The method of treatment remains debatable. The classical surgical methods include steel-wire traction and bone anchor fixation [2], and tendon-bone healing can be achieved with immobilization. We tried an innovative method by bone tunnel-tendon suture. The clinical effect and feasibility of this method was evaluated.

## Methods

From February 2017 to February 2019, 24 cases of the central slip avulsions admitted to our hospital were prospectively studied. Physical examination of these fingers showed normal passive flexion and little to no active extension at the proximal interphalangeal joint. Multiple injuries, lateral slip injury, non-insertion rupture, joint involvement, avulsion fracture and bone defect near the insertion were excluded according to the preoperative X-ray and the intraoperative visual inspection. The operation was performed on the day of injury for the open injuries. The time from injury to surgery ranged from 1 to 7 days (mean 3.6 days) for the closed injuries. All the operations were performed by the same doctor of our team.

**Fig. 1:**
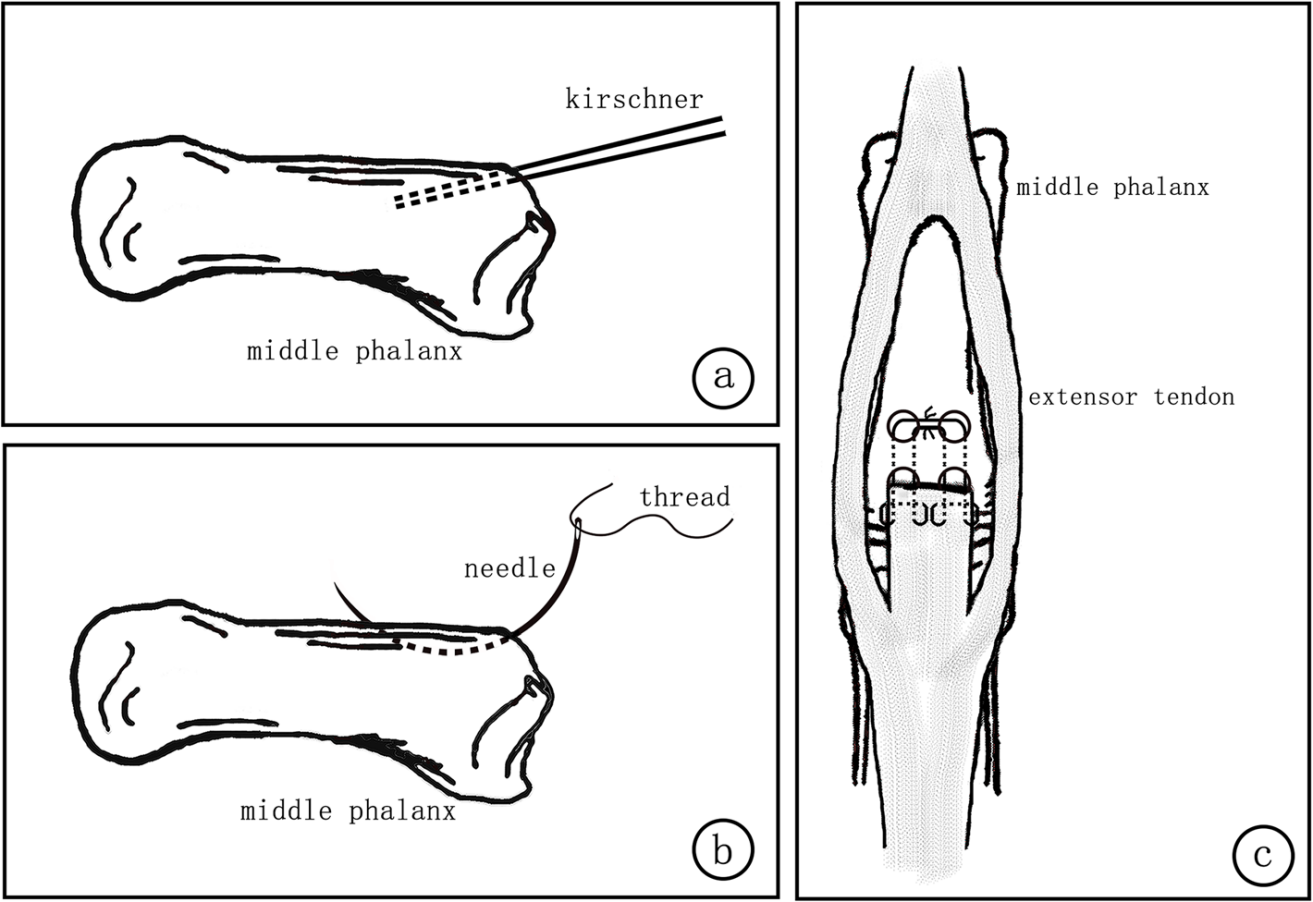
**a**-**c** Surgical schematic diagram

**Fig. 2:**
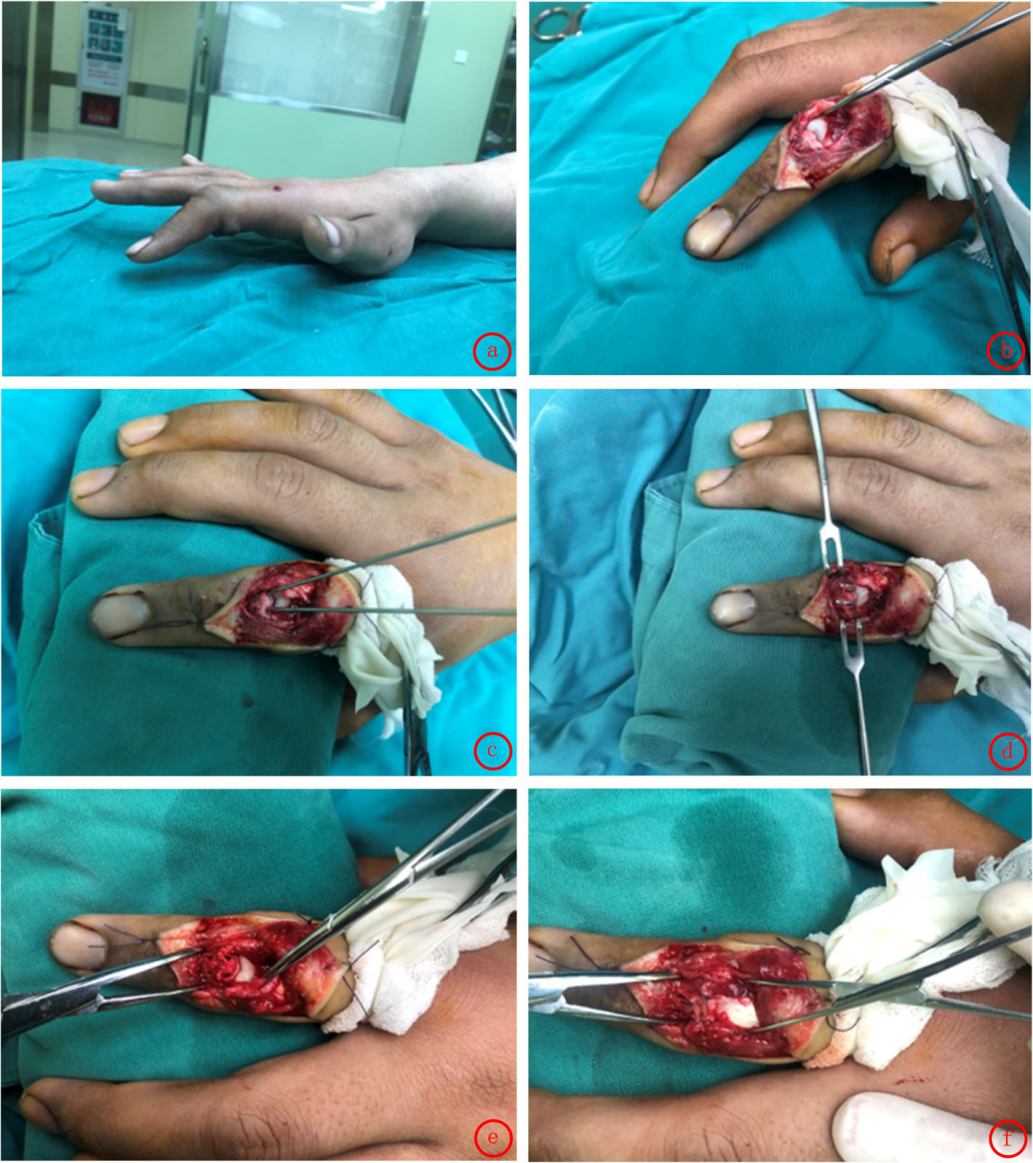
Surgical procedure: **a** limited extension, **b** exposure of central slip insertion, **c** kirschner wire drilling, **d** suture needle drilling, **e**, **f** central slip insertion repair

Under digital nerve block, a digital tourniquet at the base of the finger was applied. After the debridement in the open injuries, the original wound was extended if necessary for surgical exposure. S-shaped incision was made on the dorsum for the closed injuries. The severed central slip and its insertion site were identified. After filing the bone cortex of the insertion with the raspatory, we used the 1.2 mm kirschner wire to drill two parallel bone tunnels at the insertion point. Then, we drilled to break the dorsal side of the middle phalanx through the bone tunnels with suture needles, for the placement of 4–0 Coated VICRYL Plus sutures to anchor the central slip. The avulsed central slip was pulled to the bone tunnel. Finally, we tied the sutures respectively with the contralateral and sew up the skin incision. (Figs. 1 and 2) Without immobilization of the operated finger, active and passive functional exercise was routinely performed by the patients under the guidance of the hand therapists from the first day after surgery. The dressing was removed on the second day after surgery for better functional exercise and the incision was disinfected every day. Intravenous antibiotics were administered in the open injury cases for 2 days (on the day of surgery and the next day).

Face-to-face follow-up was conducted at 1 month, 3 months, 6 months, 12 months and 18 months after the operation. Symptoms, degree of satisfaction with the appearance, complications, Crawford’s evaluation, DASH scores and the total active movement (TAM) were collected. The range of motion (ROM) was collected within 1 year after the operation.

## Results

There were 24 cases (14 males and 10 females) included in our study with the mean age of 31.5 years (range 21 to 52). All cases were of single finger injury, involving index finger in 8, middle finger in 9, ring finger in 5 and little finger in 2 cases. There were 9 open injuries and 15 closed injuries.

All incisions or wounds healed by first intention without complications such as iatrogenic injury, infection, rerupture, pain and anchylosis. The follow-up period was 6~18 months (mean 13 months). Of 24 patients, 5 patients were followed up for less than 1 year. Telephone based follow-up was done in 2 patients revealing good recovery without complications and we couldn’t get their objective indicators.

**Fig. 3:**
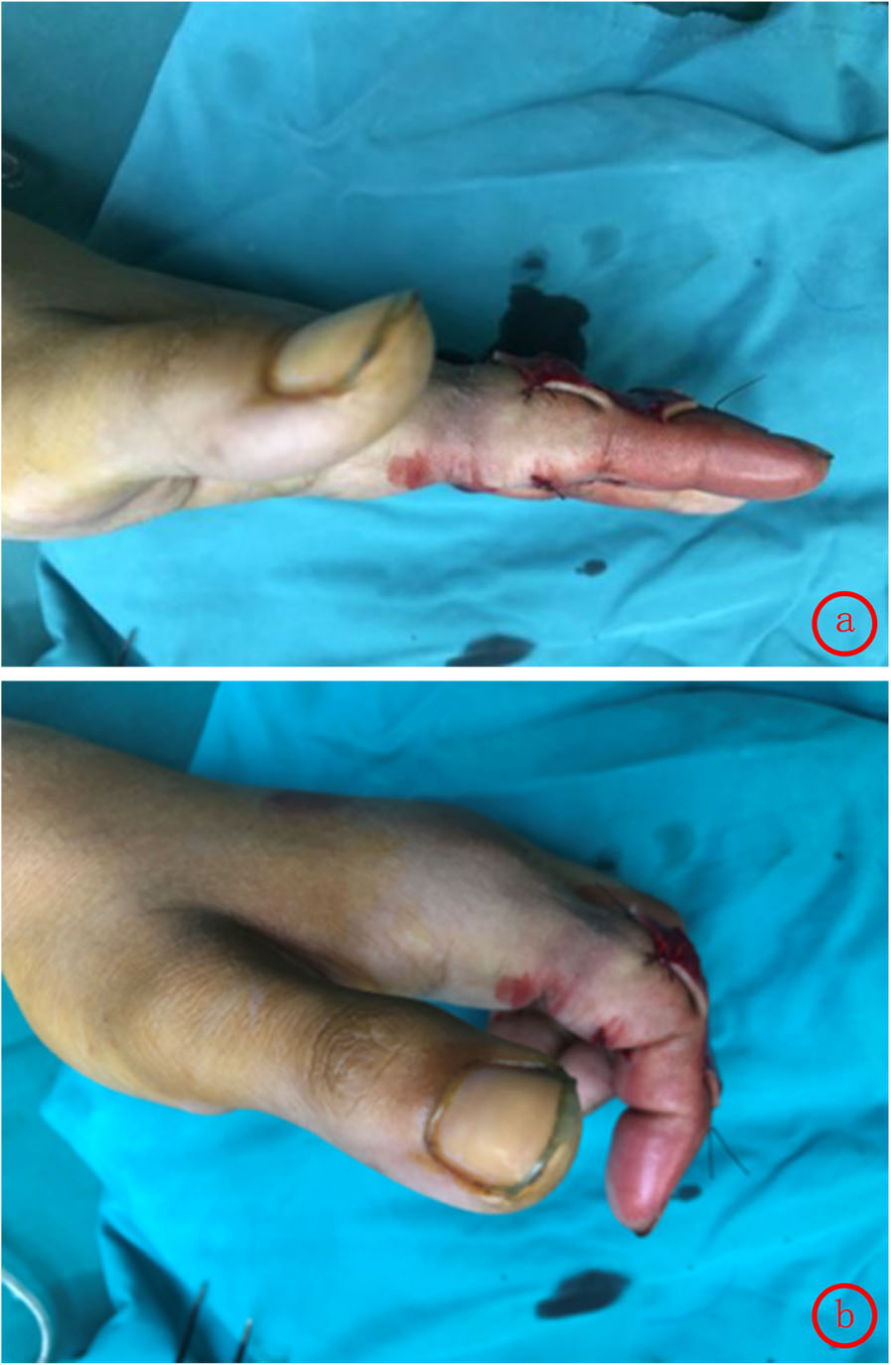
**a**, **b** Postoperative flexion and extension function

**Fig. 4:**
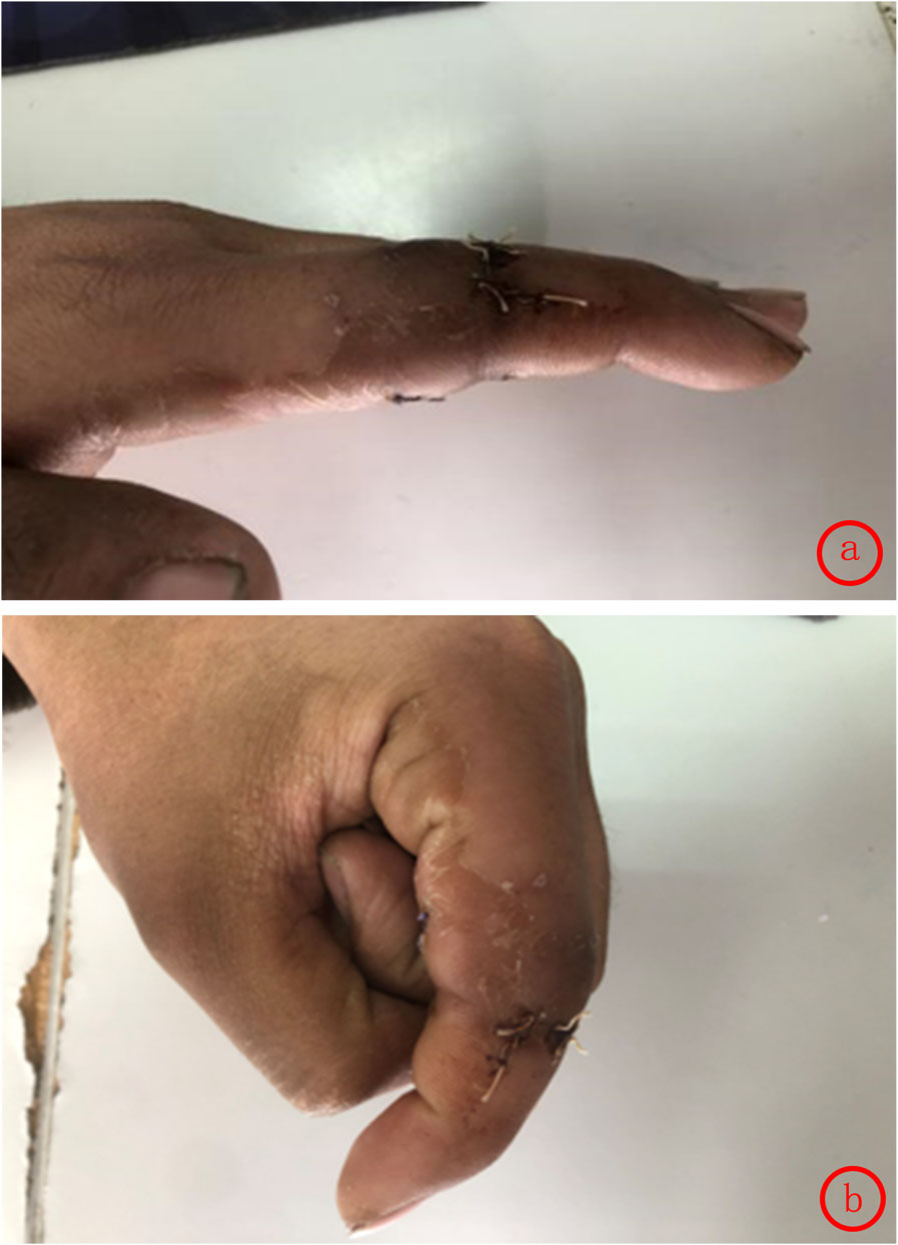
**a**, **b** Flexion and extension function at 1 month after operation

**Fig. 5:**
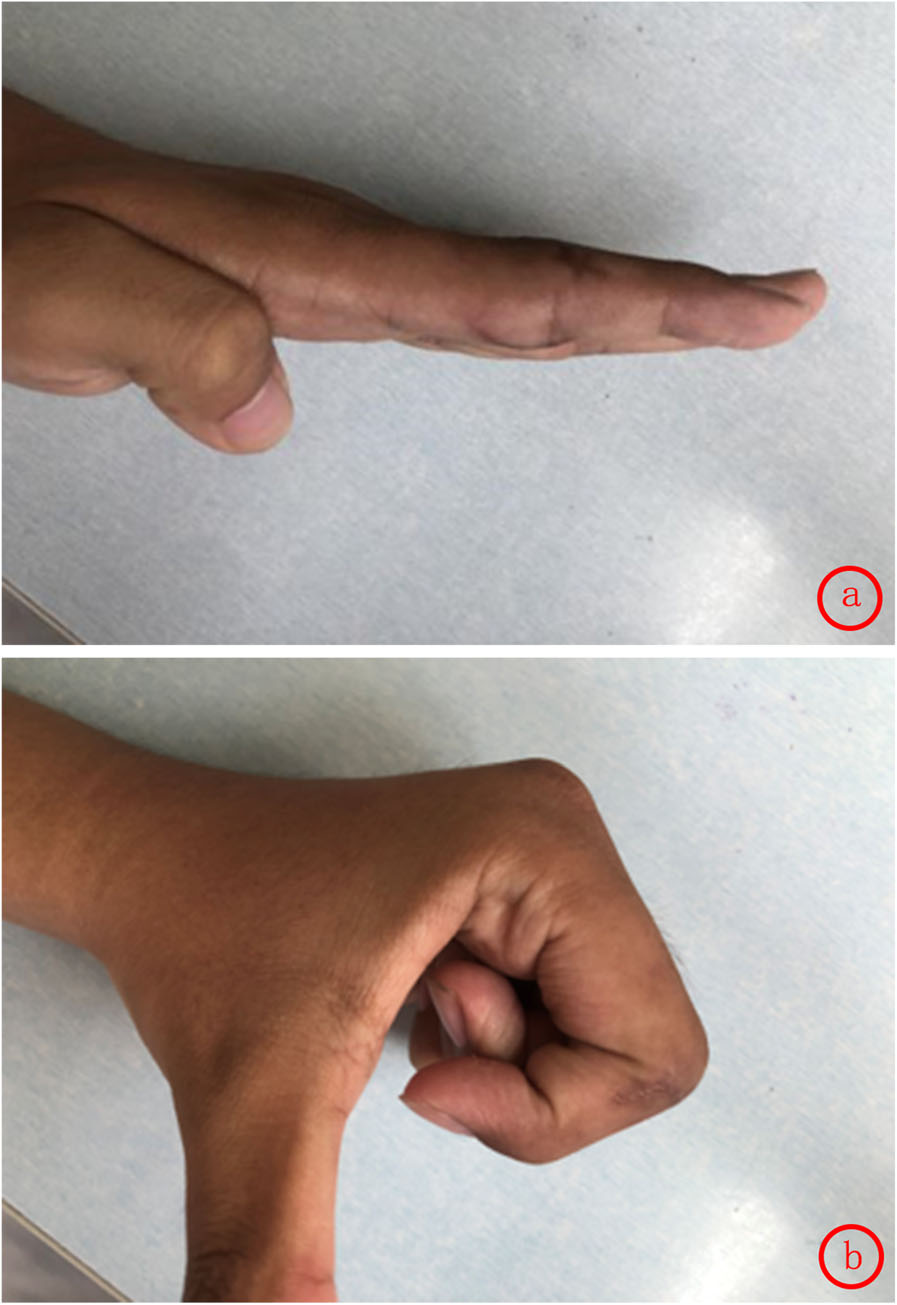
**a**, **b** Flexion and extension function at 6 months after operation

Finger function was assessed using the Crawford’s evaluation criteria [3]: excellent in 12, good in 10, average in 2, with an excellent and good rate of 91.7%. DASH [4] scores ranged from 37 to 47(mean 39). According to the Chinese Medical Association’s trial criteria for assessing the function of upper limbs^1^, excellent, good and average cases were 9, 14 and 1, respectively. In this study, standard lateral radiographs were used to determine the range of motion (ROM) of the proximal inter-phalanx in 24 patients at 1 month, 3 months and 1 year after the operation. (Table 1) There was no significant difference in the ROM deficit between the open injury group and the closed injury group at 6 months after the operation. (Table 2) The ROM gradually improved over time after the treatment, which may be related to the reduction of swelling and the functional exercise. (Figs. 3, 4, 5) The appearance evaluated by the patients was highly satisfied in 3, satisfied in 11, average in 7 and dissatisfied in 3. The cause of the dissatisfaction was continuous swelling of the joint.

**Table 1:** ROM of PIPJ during postoperative follow-up^a^

Follow-up time	One month	Three months	One year
Active extension			
Maximum	172°	182°	182°
Minimum	154°	156°	168°
Mean	158°	164°	170°
Active flexion			
Maximum	75°	90°	100°
Minimum	45°	60°	70°
Mean	56°	70°	78°

^a^*ROM* Range of motion, *PIPJ* Proximal interphalangeal joint

**Table 2:** the ROM deficit of PIPJ^a^ 6 months after the operation

Group	Open	Closed	t	*P*
The extension deficit(°)	12.1±4.0	12.7±1.3	0.562	0.580
The flexion deficit(°)	14.9±3.0	15.5±2.2	0.605	0.552

^a^*ROM* Range of motion, *PIPJ* Proximal interphalangeal joint; the ROM deficit = the unaffected side – the affected side. The ROM deficit is represented as mean±standard deviation. Two independent sample T-test is used and *P* < 0.05 is considered significantly different

Typical case: the patient was male, 24 years old, and came to hospital 4 h after crush injury of the right index finger. Physical examination showed swelling of the right index finger, skin laceration about 3 cm diagonally in the palmar side, semi-flexion of the proximal inter-phalanx, limited active extension and fine passive extension. Active extension of the distal inter-phalanx was fine. After 6 months, the appearance and functional recovery was nearly normal.

## Discussion

Green’s Hand Surgery recommends fixing for 6 weeks with the splint or kirschner wire assist for the closed injury. It is inexpensive and simple but often inadequate to fix the PIP joint in the hyperextension position making the tendon close to the insertion indirectly. Gaps between the margins lead to scar tendon healing instead of tendon-bone healing which extends the tendon length and influences the tendon function. Meanwhile, too long fixation time affects the joint motion [5] and too tight fixation may cause pressure sores.

The current evidence base for the management of central slip avulsions is limited from obtaining a best approach [6]. We believe that the treatment should first restore the tendon length and then achieve true tendon-bone healing. Therefore, in order to restore the tendon length and achieve the anatomical reduction and tendon-bone healing, surgical repair is required. The classical surgical methods include steel-wire traction and bone anchor fixation [2]. Steel-wire traction method costs less, fixes securely, but the operation is relatively complex, invasive and easy to cause collateral damage. Steel-wire traction method is not a direct reconstruction of the insertion, but a wire pull fixed on the skin surface, so that the broken tendon margin and the bone cortex contact to achieve indirect fixation with a risk of displacement and difficulty to achieve anatomical reduction. Steel-wire may influence the blood supply of tendon. The steel-wire fixed on skin surface has a great influence on the aesthetics and limits the functional exercise. Wire traction method has more risk of infection and long-term pressure may cause discomfort in patients. Severe cases may suffer from pressure sores. Some patients also fear that extraction of the wire may cause injury to the healed tendon. Sometimes in order to achieve solid fixation, the kirschner wire assist fixation may be required, which may aggravate the above shortcomings. Therefore, this method is seldom adopted clinically. Bone anchor fixation is simple and firm, but not popular because of relatively high cost and risk of loosening. Furthermore, steel-wire traction and bone anchor fixation require immobilization, which affects the functional exercise and postoperative recovery.

Many scholars have lucubrated central slip reconstruction and even the treatment of boutonniere deformity, and proposed their own strategies [7]. Some reconstructed the function of the central slip by transplanting the flexor digitorum superficialis slip [8]. This has a good effect on the central slip defect and can be applied to most cases because of few restrictions, but the original anatomical structure is changed and the damage is great. Through comparison, Ying Li et al. reported that proximal turndown of central slip combined with suture of lateral bands is superior to the free tendon grafting in terms of ROM [9].

In theory, the surface reconstruction is better than the linear reconstruction, and the linear reconstruction is better than the point reconstruction. However, the point reconstruction is often used clinically such as steel-wire traction and bone anchor fixation. In practice, it is difficult to reconstruct the surface in such a small area of the base of the middle phalanx. Therefore, our method adopts the linear reconstruction. Compared with the point reconstruction, the linear reconstruction increases the contact area of tendon-bone healing, which is theoretically beneficial to the speed and strength of healing and the recovery of tendon function.

Through bone tunnel reconstruction of the insertion, we can achieve good prognosis and anatomical reconstruction. Because of being directly sutured with the bone cortex, the central slip is not easy to dis-associate and the tendon-bone healing is improved. Without bloated external fixation, functional exercise can be performed on the first day after surgery because of solid fixation, which greatly reduces postoperative complications and speeds up postoperative recovery. Therefore, this method can also be used in specific populations such as laborers. The operation is simple and the damage is little. Almost no injury and scar union remain after healing. No secondary operation is required to extract the wire, which relieves the patient’s fear and avoids the re-injury to the tendon. It costs little and is easy to popularize. Two Kessler sutures guarantee the solid linear reconstruction.

There are some shortcomings in this surgical method. To achieve anatomical reduction and secure fixation, the bone tunnel is as parallel to the bone cortex as possible, so there is a risk of fracture when the bone tunnel is drilled with the kirschner wire and suture needles. Our follow-up time is short, the long-term effect is not clear, and the further in-depth study is needed. The cases with bone defect of the phalanx is not suitable for the above method. We need to further explore how to reconstruct the insertion in the cases with bone defect.

## Conclusions

The anatomical reconstruction of fresh central slip avulsion obtained through bone tunnel-tendon suture method, has several advantages including good prognosis, low cost, solid fixation, good range of motion, less complications and fast recovery.

## Data Availability

All data generated or analysed during this study are included in this published article.

## Abbreviations

ROM: The range of motion
TAM: The total active movement
PIP: The proximal interphalangeal joint

## Footnotes

1 Chinese Medical Association’s trial criteria for assessing the function of upper limbs is a series of criteria that assess the postoperative recovery of various upper limb injuries. The trial criteria assessed the tendon by using the total active movement (TAM) system recommended by the American Hand Surgery Association in 1975.
